# Using the Scoliometer and a Surface Topography Apparatus to Check if Back Trunk Asymmetry Changes in Children and Adolescents in the Forward Flexion and Standing Erect Positions

**DOI:** 10.7759/cureus.6334

**Published:** 2019-12-10

**Authors:** Emmanouil G Maragkoudakis, Ioannis Gelalis, Theodoros Grivas, Geofrey R Burwell, Christina Mazioti, Gerasimos Tsilimidos

**Affiliations:** 1 Orthopaedics, Private Practice, Athens, GRC; 2 Orthopaedics, University Hospital of Ioannina, Ioannina, GRC; 3 Orthopaedics and Traumatology, Tzaneio General Hospital of Piraeus, Piraeus, GRC; 4 Orthopaedics, Centre for Spinal Studies and Surgery, Nottingham University Hospitals Trust, Nottingham, GBR; 5 Scoliosis Assessment, "Tzaneio" General Hospital of Piraeus, Piraeus, GRC; 6 Family Medicine, Scoliosis Assessment, "Tzaneio" General Hospital of Piraeus, Piraeus, GRC

**Keywords:** scoliosis, assessment, scoliometer, formetrics 4d, forward flexion, standing position

## Abstract

Background

The purpose of this study is to evaluate the effects of the forward bending (FB) test versus the standing erect (SE) position on back trunk asymmetry (TA). The Scoliometer in the FB position and the 4D Formetric (4DF; Diers International, Schlangenbad, Germany) readings in the SE position were assessed.

Method

The angle of trunk inclination (ATI) was measured at the midthoracic, thoracolumbar, and lumbar levels using the Scoliometer in the FB position and the 4DF in the SE position.

A total of 134 subjects attending the scoliosis clinic (86 girls and 48 boys), age ranging from seven to 18 years, were assessed. The children and adolescents were divided into three groups according to the severity of TA, symmetric group 1 (0-2 degrees), asymmetry group 2 (2 to 6 degrees), and group 3 having asymmetry of seven or more degrees. Children with leg length discrepancy were excluded from the study.

The IBM SPSS v.20 package (IBM Corp., Armonk, NY) was used for analysis.

Results

At the midthoracic level comparing the Scoliometer to 4DF readings in males in group 1, the Wilcoxon signed ranks test was p=0.451 while for the Spearman’s Rho, it was -0.138; in group 2, p=0.184 and Rho=0.204; and in group 3, p=0.109 and Rho=0.500. For females in group 1, p=0.000 while Rho=0.003; in group 2, p=0.008 and Rho=0.000, and in group 3, p=0.003 while Rho=0.642.

At the thoracolumbar level in males for group 1, p=0.004 and Rho=-0.517; in group 2, p=0.006 and Rho=0.000; and in group 3, p=0.043 while Spearman’s Rho=0.053. For females in group 1, p=0.000 and Rho=-0.095; in group 2, p=0.000 and Rho=-0.171; in group 3, p=0.001 while Rho= -0.081. At the lumbar level for males in group 1 p=0.000 while Rho=0.149; in group 2, p=0.003 and Rho=0.373; while in group 3, p=0.109 and Rho= (-). For females in group 1, p=0.000 while Rho=-0.072; in group 2, p=0.001 and Rho=0.168; and in group 3, p=0.068 while Rho=0.500.

Conclusion

The results of this study show that the back TA in children and adolescents is not similar in the FB and SE positions. This phenomenon probably is attributed to the complicated trunkal (spinal, thoracic, and pelvic) anatomy, and the results of this study may be used as a useful foundation for further understanding of torso dynamics.

## Introduction

The purpose of this study is to evaluate the effects of the forward bending test versus the standing erect position on back trunk asymmetry [1]. The Scoliometer and the Formetrics 4D (surface topography apparatus; Diers International, Schlangenbad, Germany) readings in both the forward bending and standing erect positions of 134 examined subjects attending our scoliosis clinic (86 girls and 48 boys), age ranging from seven to 18 years, were studied.

This is an original study, which was presented at the 13th International Conference on Conservative Management of Spinal Deformities and First Joint Meeting of the International Research Society on Spinal Deformities and the Society on Scoliosis Orthopaedic and Rehabilitation Treatment - SOSORT-IRSSD 2016 meeting, Banff, Canada, 25-28 May 2016 as an oral presentation by the authors.

## Materials and methods

**Figure 1:**
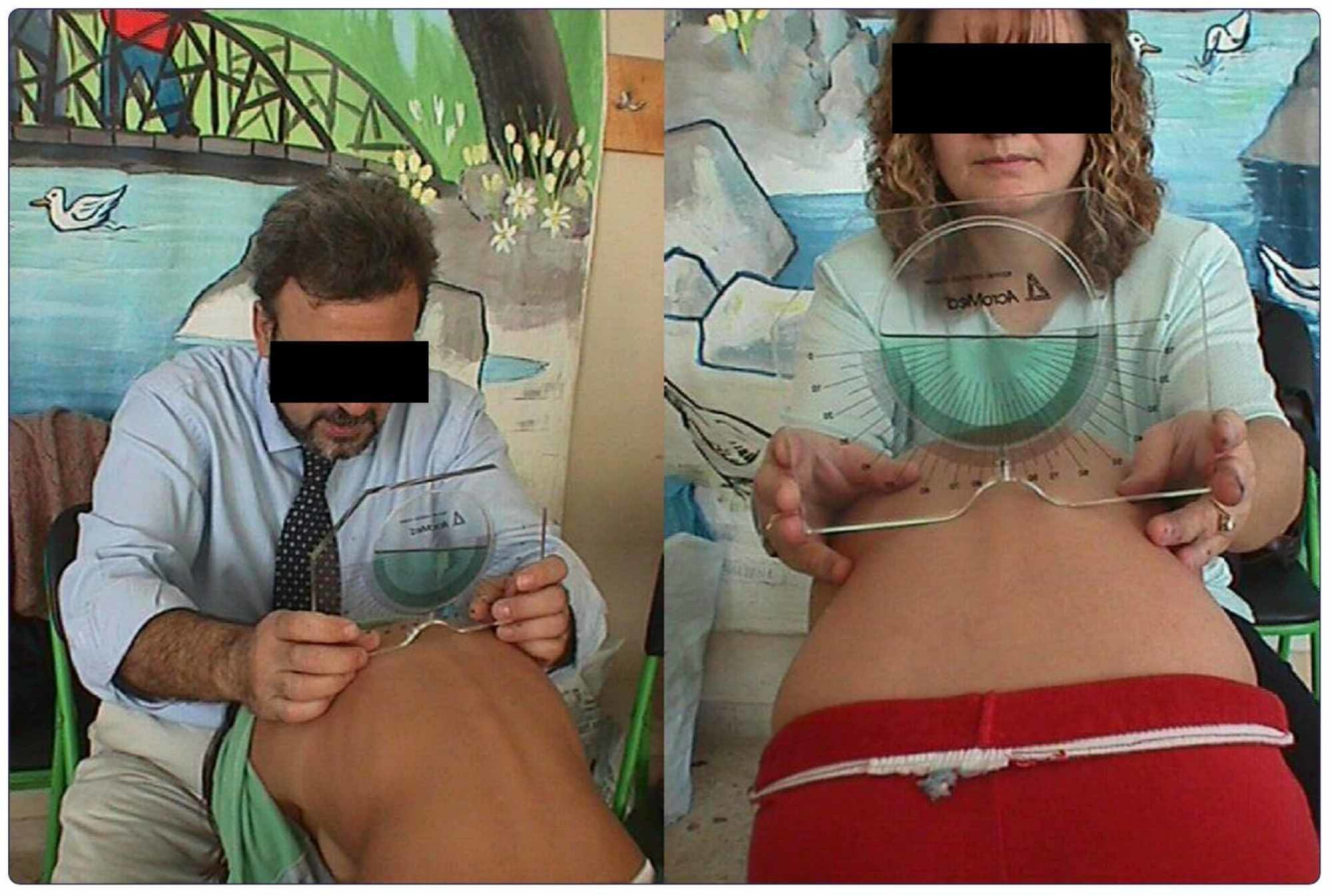
Evaluating trunk asymmetry in adolescents with idiopathic scoliosis using the Scoliometer

**Figure 2:**
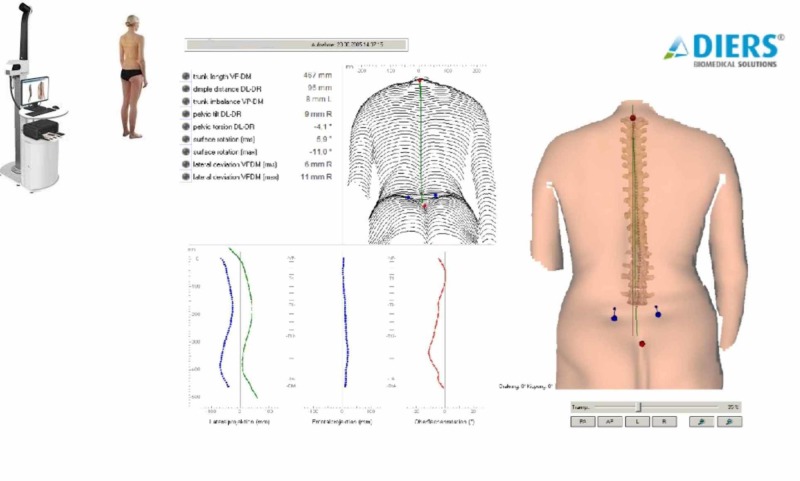
Evaluating trunk asymmetry in adolescents with idiopathic scoliosis using the Diers Formetrics 4D system Diers International, Schlangenbad, Germany

The angle of trunk inclination was measured at the midthoracic, thoracolumbar, and lumbar levels using the Scoliometer (Figure 1) and the surface topography method [1-3]. In the standing erect position, using the Formetrics system (Figure 2), the vertebral rotation, the kyphotic and lordotic angle, the pelvic obliquity and pelvic torsion, and, finally, the apical deviation were also calculated. The children and the adolescents were divided into three groups according to the severity of trunk asymmetry [4-7].

In the first group, the examined subjects were symmetric (0º-2º). In the second group, the asymmetry was two to six degrees, and in the third group, it was seven or more degrees.

For the statistical analysis, IBM SPSS v.20 (Armonk, NY) was used, calculating p-value using the Wilcoxon signed ranks test and Spearman’s Rho was used correlation coefficiency. Statistical significance (p) was set at a value of less than 0.05.

## Results

Tables 1-2 show the frequency of asymmetry in boys and girls, respectively.

**Table 1:** The mean frequency of asymmetry in the three groups in boys

Asymmetry	Scoliometer™️	Formetric™️
0-2º	37%	28%
2-6º	38%	35%
7º or >7º	23%	37%

**Table 2:** The mean frequency of asymmetry in the three groups in girls

Asymmetry	Scoliometer™️	Formetric™️
0-2º	63%	72%
2-6º	62%	65%
7º or >7º	77%	63%

In our sample, the mean frequency of symmetry (0º-2º) in boys and in girls was 37% and 63%, respectively, using the Scoliometer and 28%-72% using the Formetrics 4D. The mean frequency of asymmetry (2º-6º) for the boys was 38%, and for the girls, it was 62% using the Scoliometer and 35% for boys and 65% for girls using Formetrics 4D. The mean frequency of asymmetry of 7º or more was 23% for the boys and 77% for the girls using the Scoliometer and 37% for boys and 63% for girls using Formetrics 4D.

**Figure 3:**
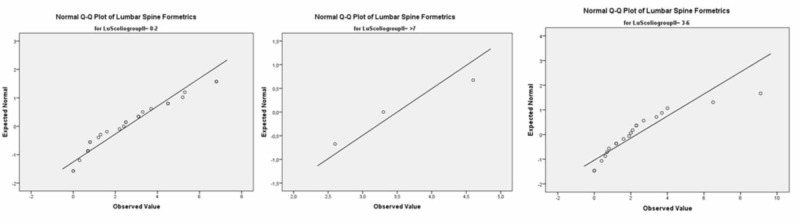
Male groups 1, 2, 3 at the midthoracic level (Q-Q Plot)

At the midthoracic spinal level in group 1 (0º-2º), for males, the p-value was 0.451 while Spearman’s Rho for the correlation coefficiency was -0.138. In group 2 (2º-6º), for males, the p-value was 0.184 and Spearman’s Rho for the correlation coefficiency was 0.204. For the males in group 3 (7º+), the p-value was 0.109 while Spearman’s Rho for the correlation coefficiency was 0.500. See Figure 3.

**Figure 4:**
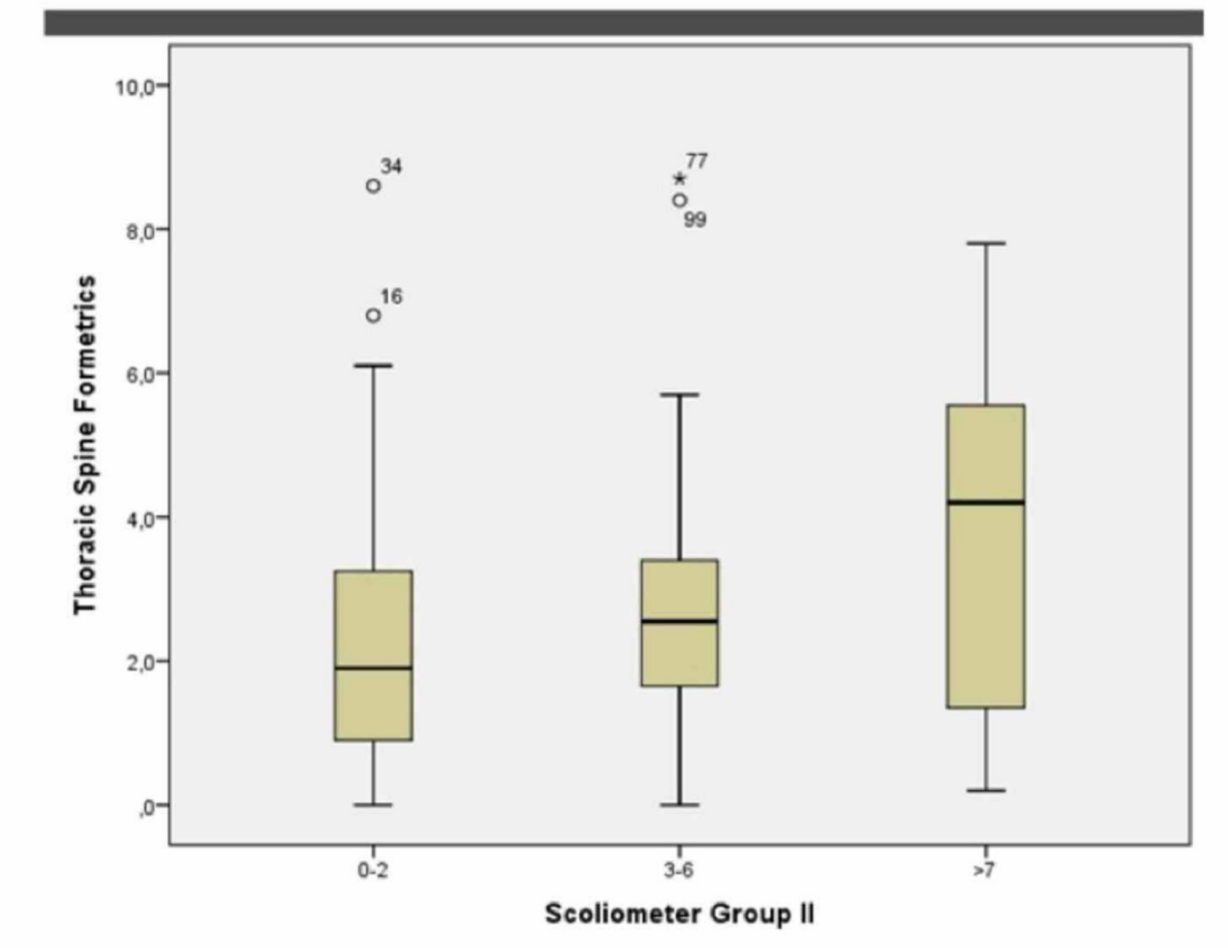
Female groups 1, 2, 3 at the midthoracic level (Boxplot)

At the midthoracic level in group 1 (0º-2º), for females, the p-value was 0.000 while Spearman’s Rho for the correlation coefficiency was 0.003. In group 2 (2º-6º), the p-value was 0.008 while Spearman’s Rho for the correlation coefficiency was 0.000. In group 3 (7º+), the p-value was 0.003 while Spearman’s Rho for the correlation coefficiency was 0.642. See Figure 4.

**Figure 5:**
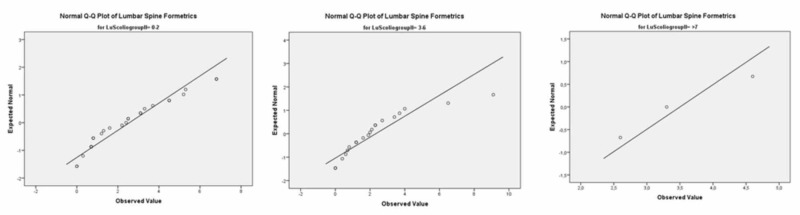
Male groups 1, 2, 3 at the thoracolumbar level (Q-Q Plot)

At the thoracolumbar spinal level in group 1 (0º-2º), for males, the p-value was 0.004 while Spearman’s Rho for the correlation coefficiency was -0.517. In group 2 (2º-6º), the p-value was 0.006 and Spearman’s Rho for the correlation coefficiency was 0.000. In group 3 (7º+), the p=value was 0.043 while Spearman’s Rho for the correlation coefficiency was 0.053. See Figure 5.

**Figure 6:**
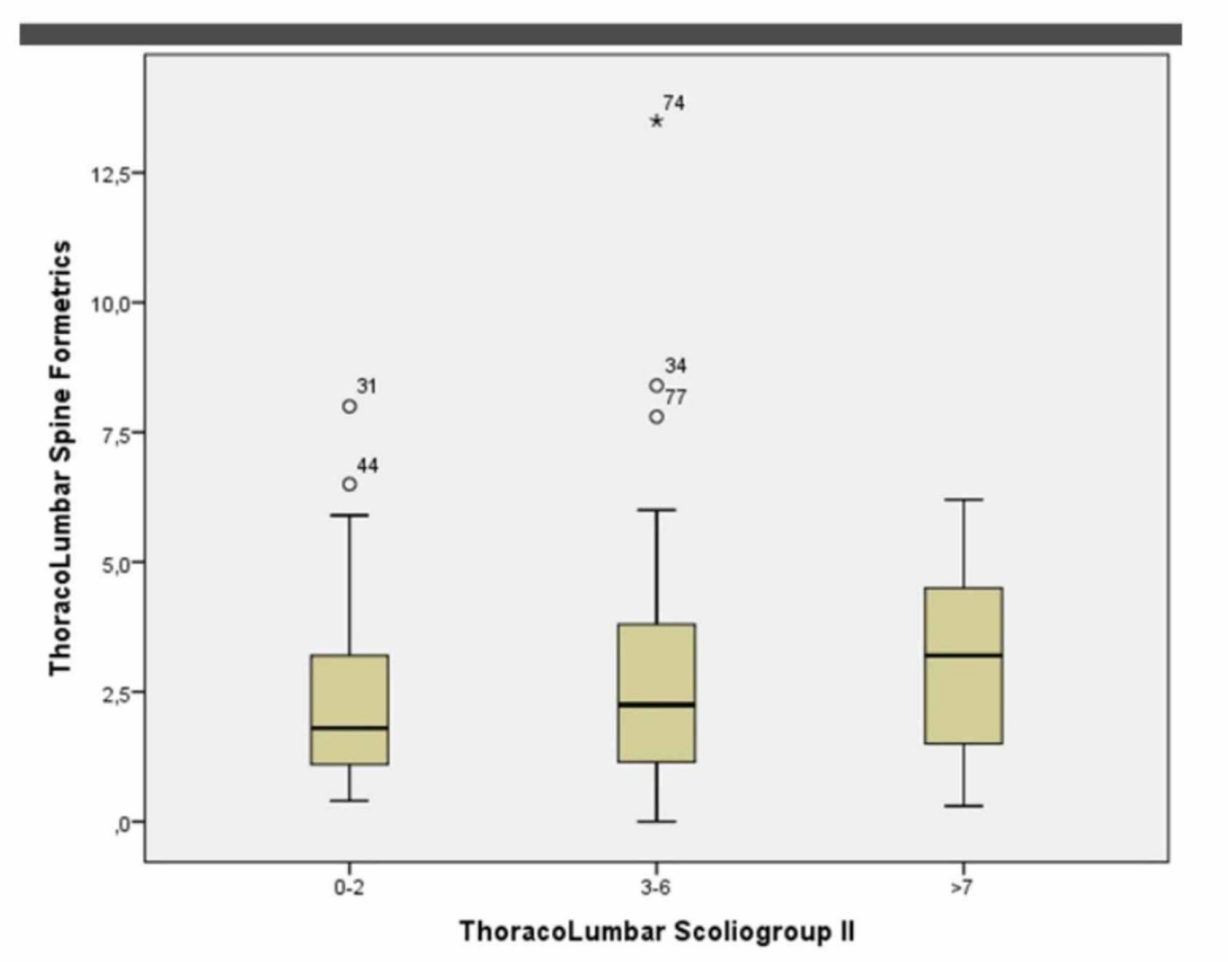
Female groups 1, 2, 3 at the thoracolumbar level (Boxplot)

At the thoracolumbar level in group 1 (0º-2º), for females, the p-value was 0.000 while Spearman’s Rho for the correlation coefficiency was -0.095. In group 2 (2º-6º), the p-value was 0.000 while Spearman’s Rho for the correlation coefficiency was -0.171. In group 3 (7º+), the p-value was 0.001 while Spearman’s Rho for the correlation coefficiency was -0.081. See Figure 6.

**Figure 7:**
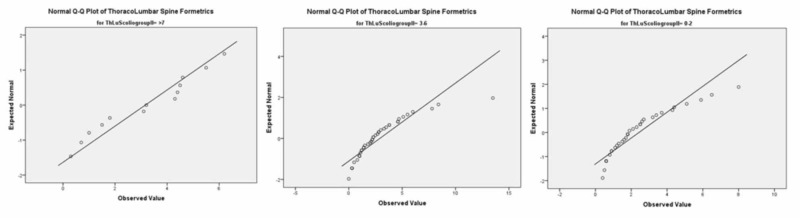
Male groups 1, 2, 3 at the lumbar level (Q-Q Plot)

At the lumbar spinal level in group 1 (0º-2º), for males, the p-value was 0.000 while Spearman’s Rho for the correlation coefficiency was 0.149. In group 2 (2º-6º), the p-value was 0.003 and Spearman’s Rho for the correlation coefficiency was 0.373. In group 3 (7º+), the p-value was 0.109 while Spearman’s Rho for the correlation coefficiency was (-). See Figure 7.

**Figure 8:**
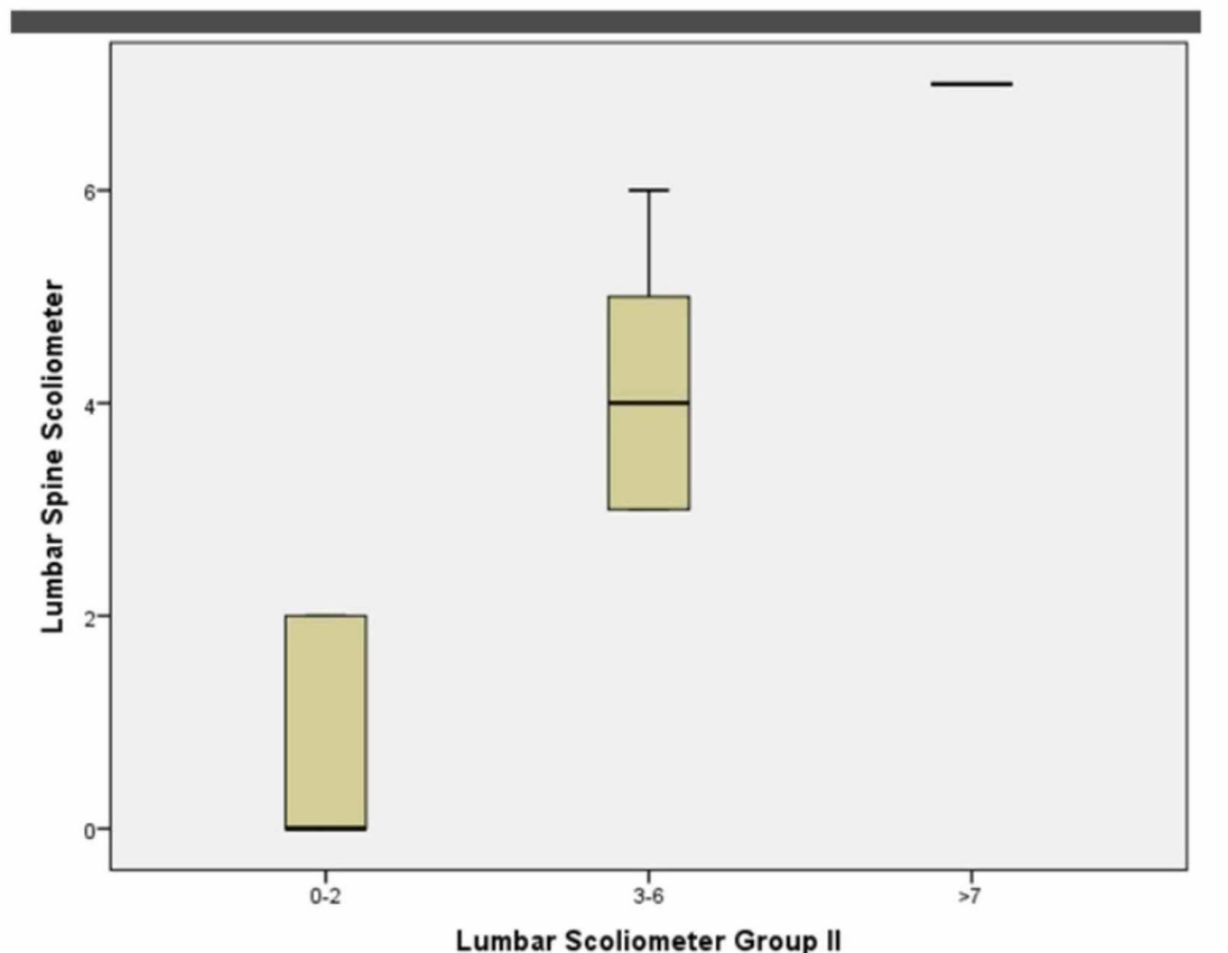
Female groups 1, 2, 3 at the lumbar level (Boxplot)

At the lumbar spinal level in group 1 (0º-2º), for females, the p-value was 0.000 while Spearman’s Rho for the correlation coefficiency was -0.072. In group 2 (2º-6º), the p-value was 0.001 while Spearman’s Rho for the correlation coefficiency was 0.168. In group 3 (7º+), the p-value was 0.068 while Spearman’s Rho for the correlation coefficiency was 0.500. See Figure 8.

## Discussion

For the past several years, there has been a great evolution of the technology and knowledge of surface topography. The reliability of the measurements has been widely researched and many publications have been elaborated throughout the literature. On the other hand, the Scoliometer has been broadly used since its invention by W.P. Bunnel in 1984 during a scoliosis assessment. The key point of the measurements' comparison study is the posture of the object studied.

It is obvious that in all three anatomical regions of the spine, in both males and females, the change from a forward bending position to a standing erect position shows a reduction of the mean trunk asymmetry, probably due to the vertebral relation changes during the change of the two positions [8].

We already know from past clinical studies that the three main mechanical components of a primary structural scoliosis curve of the usual rotatory type are rotation, tilt, and lordosis [9]. The initial event is usually a failure of rotation control in the spine. This develops principally during gait due to asymmetrical forces resulting from rib-vertebra angle asymmetry, which, in turn, is caused by abnormal developmental mechanisms in the central nervous system. The changes of vertebral tilt in each of the frontal and sagittal planes of the spine are usually secondary to the vertebral rotation [10].

In 1865, Adams published the fact that vertebral rotation causes lordosis and lateral curve. At the ends of the spinal curve, the thoracic vertebrae are rotating about a normal anterior axis of rotation. In contrast, at the curve apex, the thoracic vertebrae are rotating about an abnormal posterior axis of rotation, more like a lumbar vertebra [11]. It must also be mentioned at this point that there is also the so-called intravertebral and discal torsion, which is present along the length of the spine and are minimal at the curve apex [12].

Certain muscles, through the central nervous system (sacrospinalis, iliocostalis, levator scapulae, rhomboid, serrates anterior, external and internal oblique muscles, and so on), play a role in trunk rotation and gait by elevating the upper ribs to change the rib-vertebra angles from a funnel-shaped chest at birth to a more broad-shaped thorax in adolescence. The musculature complex is the so-called spiral composite muscle trunk rotator [13].

The sagittal configuration of the spinopelvic complex (crucially different between humans and other vertebrates) has obvious consequences for its biomechanical loading, but often-used parameters like thoracic kyphosis and lumbar lordosis are relatively useless for understanding biomechanical loading since the same numerical value for kyphosis can have any different position relative to gravity. It is well-understood that in the standing erect position and in the forward bending position, all the above-mentioned characteristics of the spine and thoracic cage undergo changes, which explains the findings of this study.

The necessity of a scoliosis assessment has led to the school screening program in which students from all over the country get examined in all three anatomical regions of the spine ( thoracic - thoracolumbar - lumbar ) in both positions. There is no doubt that in the future, due to the technological evolution of the surface topography apparatuses and the broad use of computers in everyday life and in medical practice, scoliosis will be assessed both in the forward bending position and in the standing erect position.

## Conclusions

The results of this study show that the back trunk asymmetry in children and adolescents is not similar in the forward bending and standing erect positions. This phenomenon probably is attributed to the complicated trunkal (spinal, thoracic, and pelvic) anatomy, and the results of this study may be used as a useful foundation for a further understanding of torso dynamics.

## References

[1] Frerich JM, Hertzler K, Knott P, Mardjetko S (2012). Comparison of radiographic and surface topography measurements in adolescents with idiopathic scoliosis. Open Orthop J.

[2] Patias P, Grivas TB, Aggouris C, Drakoutos E (2010). A review of the trunk surface metrics used as Scoliosis and other deformities evaluation indices. Scoliosis.

[3] Knott P, Mardjetko S, Rollet M, Baute S, Riemenschneider M, Muncie L (2010). Evaluation of the reproducibility of the formetric 4D measurements for scoliosis. Scoliosis.

[4] Mangone M, Raimondi P, Paoloni M (2013). Vertebral rotation in adolescent idiopathic scoliosis calculated by radiograph and back surface analysis-based methods: correlation between the Raimondi method and rasterstereography. Eur Spine J.

[5] Diers H, Mooshake S, Heitmann KR (2009). Dynamic 3D (4D) in objective classification of severe back deformities. Scoliosis.

[6] Raso VJ, Lou E, Hill DL, Mahood JK, Moreau MJ, Durdle NG (1998). Trunk distortion in adolescent idiopathic scoliosis. J Pediatr Orthop.

[7] Melvin M, Sylvia M, Udo W, Helmut S, Paletta JR, Skwara A (2010). Reproducibility of rasterstereography for kyphotic and lordotic angles, trunk length, and trunk inclination. A reliability study. Spine (Phila Pa 1976).

[8] Burwell RG, Cole AA, Cook TA (1992). Pathogenesis of idiopathic scoliosis. The Nottingham concept. Acta Orthopaedica Belgica.

[9] Wemyss-Holden SA, Butcher CA, Burwell RG, Webb JK, Moulton A (1991). Segmental evaluation of the rotational (torsional) deformity in scoliosis: clinics-anatomical observations and surgical significance. Clin Anat.

[10] Cook TA, Burwell RG, Wemyss-Holden SA, Webb JK (1991). A segmental study of iliocostalis: functional and clinical implications. Clin Anat.

[11] Wemyss-Holden SA, Burwell RG, Cook TA, Binch C, Webb JK, Moulton A (1990). A spiral composite trunk rotator in man. Relevance to gait, idiopathic and sportsman’s scoliosis and stroke. Clin Anat.

[12] Keller TS, Colloca CJ, Harrison DE, Harrison DD, Janik TJ (2005). Influence of spine morphology on intervertebral disc loads and stresses in asymptomatic adults: implications for the ideal spine. Spine J.

[13] Schlosser TP, Vincken KL, Rogers K, Castelein RM, Shah SA (2014). Natural sagittal spino-pelvic alignment in boys and girls before, at and after the adolescent growth spurt. Eur Spine J.

